# The HIV epidemic and the COVID-19 pandemic: A double tragedy for sub-Saharan African women

**DOI:** 10.4102/phcfm.v14i1.3397

**Published:** 2022-08-26

**Authors:** Grant Murewanhema, Godfrey Musuka, Knowledge Denhere, Delarise Mulqueeny, Tafadzwa Dzinamarira

**Affiliations:** 1 Department of Primary Health Care Sciences, Faculty of Medicine, University of Zimbabwe, Harare, Zimbabwe; 2 ICAP, Columbia University, Harare, Zimbabwe; 3 School of Public Health, University of the Western Cape, Cape Town, South Africa; 4 Department of Social Work, University of Zululand, Richards Bay, South Africa; 5 Department of Health Systems and Public Health, Faculty of Health Sciences, University of Pretoria, Pretoria, South Africa

**Keywords:** HIV, COVID-19, women, sub-Saharan Africa, disease burden

## Abstract

After four decades of the HIV epidemic, women from sub-Saharan Africa remain at a differentially high risk of acquisition. The Joint United Nations Programme on HIV and AIDS (UNAIDS) statistics show that the majority of HIV infections occur in this population and region. Evidence from previous humanitarian crises demonstrated adverse maternal consequences as a result of neglect for the provision of essential maternal, sexual and reproductive health services. The ongoing COVID-19 pandemic has had a similar effect, including an additional risk of HIV acquisition amongst women in sub-Saharan Africa. The COVID-19 pandemic has aggravated the risk of sub-Saharan Africa women to HIV infection because of a multitude of factors including child marriages, teenage pregnancies, dropping out of school, increase in incidence of sexual and gender-based violence and reduced access to preventive and treatment services for HIV and sexually transmitted infections. These include provision of care for rape and sexual and gender-based violence victims and provision of pre-exposure and postexposure prophylaxis for HIV and other STIs. Failure to urgently restore and maintain robust HIV prevention and treatment during the ongoing COVID-19 pandemic poses a risk of reversing the gains made over the years in reducing the incidence and morbidity from HIV amongst the population of sub-Saharan Africa women. There is need for an urgent and robust discourse to formulate effective interventions for protecting women and girls living in sub-Saharan Africa from an aggravated risk of HIV infection during the ongoing COVID-19 pandemic and other future humanitarian crises.

## Background

Despite substantial progress in the reduction of human immunodeficiency virus (HIV) incidence, young women from sub-Saharan Africa (SSA) remain at a substantial risk of HIV infections.^1^ The World Health Organization (WHO) defines significant risk as an incidence of HIV infection in the absence of pre-exposure prophylaxis (PrEP) that is sufficiently high (> 3% incidence). The Joint United Nations Programme on HIV/AIDS (UNAIDS) statistics estimate that close to 60% of new infections occur amongst young women, with an estimated 5500 young women acquiring HIV weekly.^2^ Sub-Saharan Africa accounts for 67% of these incidents of infection. The search for an effective HIV vaccine still remains elusive after decades of trials and research, resulting in behavioural and biomedical interventions remaining critical over the years.^3^ These interventions include encouraging the negotiation and practice of safer sex, reducing the number of sexual partners, seeking early and appropriate treatment for sexually transmitted infections (STIs) and the use of PrEP. Untreated ulcerative and nonulcerative STIs significantly increase the risk of HIV infections severalfold. The approval of different PrEP formulations is a welcome development, as they provide women with methods they can have control over in predominantly patriarchal African societies.

HIV prevention and treatment programmes in numerous SSA countries are supported by development partners, sometimes with limited support and funding from local governments.^4^ In addition, the over-reliance on unilateral aid is often conceptualized in global health as a ticking time bomb. The ongoing coronavirus disease 2019 (COVID-19) pandemic, which has far-reaching public health implications and consequences, may negatively impact on the provision of both preventative and treatment services for HIV in SSA countries, thereby putting them at an additional risk of HIV acquisition. Many organisations that support HIV prevention and treatment programmes, alongside mainstream governments, shifted their priorities, responses and resources to COVID-19 responses, resulting in certain areas of public health, such as maternal, sexual and reproductive health being neglected, as the primary focus was shifted to COVID-19 containment and mitigation.^5^

Evidence from previous humanitarian crises has shown the aggravated vulnerability of women to adverse sexual and reproductive health (SRH) outcomes.^6^ This is evident in the Ebola virus disease outbreaks in West Africa, where significant rises in maternal mortality were recorded as women failed to timeously and adequately access these services.^7^ Despite substantial lessons from the past, little is implemented to protect young girls and women from the negative effects of natural and health disasters on the provision of essential SRH services. Healthcare systems have been overwhelmed by the demands that the COVID-19 pandemic have presented, with most countries failing to maintain SRH amenities, resulting in neglect and an increase in maternal ill-health.^8^ This is demonstrated by the increase in maternal deaths in SSA by between 8% and 39% as a result of the direct effects of COVID-19 on pregnant women.^9^ Economic recessions, which are more pronounced in SSA, contribute to loss of income for families, often resulting in women being exposed to high-risk jobs such as vending, transactional sex, cross-border trading and commercial sex. An increase in sexual exploitation and gender-based violence (GBV) has been reported in Uganda.^10^ Sexual and gender-based violence (SGBV) also exposes women to additional risks of HIV infection through forced, dry, unprotected penetrative anal and oral sex.

The COVID-19 pandemic has exacerbated the prevailing gender inequalities in economic prospects in SSA, with studies showing an increase in the number of child marriages and early pregnancies as the female child becomes a source of quick income for their families. Prolonged school closures have also contributed to increased school dropout rates, teenage pregnancies, child marriages and the risk of young girls and women ending up in polygamous marriages as a result of increased poverty and social vulnerability.^11,12^ In Zimbabwe, child marriage is still rife and bride-price is the norm within certain religious sects.^12^ This includes the apostolic movement, which promotes polygamy and child marriage. This movement also discourages the use of condoms and modern contraception.^12^ This contributes to women experiencing a multitude of challenges discussed here.

A study on the hardest-to-reach, rural adolescent girls in Rwanda, Uganda and Tanzania found that because of COVID-19, adolescent girls had turned to and engaged in transactional sex to afford and provide for their basic needs, with approximately 30% dropping out of school for pregnancy-related reasons.^8^ A study on adolescent girls in Kenya reported that COVID-19 had amplified young women’s monetary dependence on transactional sex by an estimated 50%.^13^ In Rwanda, less than half of women living with HIV attended antiretroviral therapy (ART) collection clinic appointments in the first month of the COVID-19 lockdown.^14^ A similar pattern was observed in the number of women attending prevention of mother-to-child transmission (PMTCT) of HIV services in the first few months of the COVID-19 lockdown in Uganda.^15^

The ongoing COVID-19 pandemic is widely reported to have enhanced the risk of women to adverse SRH outcomes, including the risk of HIV and human papilloma virus (HPV) acquisitions. The COVID-19 pandemic has multiplied the risk of HIV acquisition for young women by aggravating pre-existing risk factors. Closure of rape and SGBV clinics in some countries has resulted in women victims requiring access to STI prophylaxis, emergency contraception and HIV postexposure prophylaxis (PEP) medicines. The safety nets that usually protect women from social ills are obscured during times of pandemics, with access to prevention services and treatment for STIs and HIV being reduced. Hence, victims of SGBV fail to timeously access HIV testing, emergency contraception, STI prophylaxis and HIV PEP. Amnesty International has highlighted the risk of security officers abusing young women and girls during crisis times, with young women being exposed with nowhere to turn for assistance.

## Conclusion

The COVID-19 pandemic has been at the centre stage of the world’s attention for the past 2 years, and it is now evident that the battle with the pandemic will be protracted, with periods of low transmission and intervening harsh waves. Though countries are making substantial progress with vaccinations, the risks of further restrictions, disruptions in global supply chains of essential health commodities, loss of jobs and school interruptions are likely to persist. Gatekeepers, global players and relevant public health stakeholders in SRH involved in the protection of women, girls and vulnerable populations must be cognizant of the double tragedy confronting women living in HIV and COVID-19 transmitting locations such as SSA. Ensuring urgent restoration and maintenance of robust HIV prevention and treatment services in this region is key, making sure there are minimal disruptions henceforth. In the context of new demands on funding in African countries, the reliance on external donors and fragmented health systems, there is need for sustainable measures to protect HIV and SRH services. Innovative approaches that range from task shifting at the primary health centre level, to supporting decentralization of rape and SGBV clinics, to promoting investment through mechanisms such as public-private partnerships could prove beneficial. Though proactive strategic interventions require concerted, multidisciplinary involvement of various SRH stakeholders, we are hopeful that this short communique will serve as a useful reminder of the possibility.
